# The Cold-Adapted, Temperature-Sensitive SARS-CoV-2 Strain TS11 Is Attenuated in Syrian Hamsters and a Candidate Attenuated Vaccine

**DOI:** 10.3390/v15010095

**Published:** 2022-12-29

**Authors:** Jiayu Xu, Mingde Liu, Xiaoyu Niu, Juliette Hanson, Kwonil Jung, Peng Ru, Huolin Tu, Daniel M. Jones, Anastasia N. Vlasova, Linda J. Saif, Qiuhong Wang

**Affiliations:** 1 Center for Food Animal Health, Department of Animal Sciences, College of Food, Agricultural and Environmental Sciences, The Ohio State University, Wooster, OH 44691, USA; 2 Department of Veterinary Preventive Medicine, College of Veterinary Medicine, The Ohio State University, Columbus, OH 43210, USA; 3 The Ohio State University Comprehensive Cancer Center, The Ohio State University James Cancer Center, Columbus, OH 43210, USA; 4 James Molecular Laboratory at Polaris, The Ohio State University James Cancer Center, Columbus, OH 43240, USA; 5 Department of Pathology, The Ohio State University Wexner Medical Center, Columbus, OH 43210, USA

**Keywords:** SARS-CoV-2, COVID-19, coronavirus, vaccine, cold-adaptation, temperature-sensitive, Syrian hamster

## Abstract

Live attenuated vaccines (LAVs) replicate in the respiratory/oral mucosa, mimic natural infection, and can induce mucosal and systemic immune responses to the full repertoire of SARS-CoV-2 structural/nonstructural proteins. Generally, LAVs produce broader and more durable protection than current COVID-19 vaccines. We generated a temperature-sensitive (TS) SARS-CoV-2 mutant TS11 via cold-adaptation of the WA1 strain in Vero E6 cells. TS11 replicated at >4 Log_10_-higher titers at 32 °C than at 39 °C. TS11 has multiple mutations, including those in nsp3, a 12-amino acid-deletion spanning the furin cleavage site of the S protein and a 371-nucleotide-deletion spanning the ORF7b-ORF8 genes. We tested the pathogenicity and protective efficacy of TS11 against challenge with a heterologous virulent SARS-CoV-2 D614G strain 14B in Syrian hamsters. Hamsters were randomly assigned to mock immunization-challenge (Mock-C) and TS11 immunization-challenge (TS11-C) groups. Like the mock group, TS11-vaccinated hamsters did not show any clinical signs and continuously gained body weight. TS11 replicated well in the nasal cavity but poorly in the lungs and caused only mild lesions in the lungs. After challenge, hamsters in the Mock-C group lost weight. In contrast, the animals in the TS11-C group continued gaining weight. The virus titers in the nasal turbinates and lungs of the TS11-C group were significantly lower than those in the Mock-C group, confirming the protective effects of TS11 immunization of hamsters. Histopathological examination demonstrated that animals in the Mock-C group had severe pulmonary lesions and large amounts of viral antigens in the lungs post-challenge; however, the TS11-C group had minimal pathological changes and few viral antigen-positive cells. In summary, the TS11 mutant was attenuated and induced protection against disease after a heterologous SARS-CoV-2 challenge in Syrian hamsters.

## 1. Introduction

The severe acute respiratory syndrome coronavirus-2 (SARS-CoV-2) is the cause of coronavirus disease 2019 (COVID-19), which emerged in December 2019 in Wuhan, China [1]. The COVID-19 pandemic has resulted in over 6.6 million deaths as of 24 November 2022 according to the World Health Organization. Different types of COVID-19 vaccines, including the viral spike (S) protein-based mRNA vaccines, adenovirus-vectored vaccines and inactivated vaccines, have been developed, used and have contributed to reduced disease severity and mortality in many countries [2]. Two mRNA vaccines (BioNTech and Pfizer and Moderna) and one adenovirus type 26 (Ad26) vectored vaccine (Johnson & Johnson) have been authorized for emergency use by the US Food and Drug Administration. In the United States, 228 million people have completed the primary series of vaccination (https://COVID.cdc.gov/COVID-data-tracker/#vaccinations (accessed on 30 November 2022)) as of 24 November 2022, and the vaccination campaign using the updated Bivalent vaccines, which contain the components of the original SARS-CoV-2 strain and Omicron variant, is still ongoing. However, in many low- and middle-income countries (LMICs), COVID-19 vaccination rates are substantially lower, partially due to the limitations of existing cold chains for the storage and distribution of vaccines, especially the mRNA vaccines that require ultracold temperatures [3].

In addition, evidence has shown that the S protein-based vaccines offer limited protection against infection with emerging SARS-CoV-2 variants. For example, the most recent Omicron variant has escaped the primary strain-based vaccine-induced immunity and quickly spread worldwide [4]. In contrast, natural SARS-CoV-2 infection can prevent around 90% reinfection with the Alpha, Beta or Delta variants of concern (VOC) and 56% reinfection with the Omicron VOC, and most reinfections occurred after one year [5]. Clinical and experimental studies also emphasized the importance of protective mucosal immune responses induced by natural SARS-CoV-2 infection (but not COVID-19 vaccination) that can prevent re-infection of most people for at least 6 months [6,7]. Another advantage of the immunity induced by natural infection is the fact that SARS-CoV-2 expresses 16 non-structural proteins (nsp1-nsp16), four structural proteins [S, membrane (M), envelop (E), and nucleocapsid (N)] and nine accessory proteins [1] that can independently evoke and shape the immune responses. The M and N structural proteins are highly immunogenic and they and the non-structural proteins share a higher sequence identity than the S protein among different SARS-CoV-2 variants. Additionally, many conserved T cell epitopes to genetically related CoVs and SARS-CoV-2 variants are present on non-S proteins [8,9]. Coordinated T cell responses induced by natural infection correlated with mild COVID-19 cases, while delayed innate immune responses were associated with severe disease [10]. Furthermore, T cell responses were not considerably affected by mutations found in the SARS-CoV-2 VOC [11,12]. Therefore, T cell epitopes have the potential to broaden and prolong protection against emerging variants. In addition, viral peptides presented by the class I human leukocyte antigen (HLA-I) are derived not only from canonical ORFs but also from alternative ORFs in the S gene and N gene, which are not included in current vaccines [13]. Live attenuated vaccines (LAVs) contain all the viral essential protein genes and they are capable of replication in the respiratory mucosa, mimicking natural infection. Thus, LAVs presumably induce broader and more balanced mucosal and systemic humoral and T cell responses than other types of vaccines. LAVs can be used for primary vaccination followed by boosting with the adenovirus-vectored, inactivated virus, or S mRNA/protein-based vaccines to induce broader and more durable immunity as evident when SARS-CoV-2 booster vaccines are given to infected-and-recovered individuals [14]. An additional practical advantage of LAVs is easier (intranasal) administration associated with increased public acceptance than intramuscular vaccines, including mRNA vaccines. In summary, LAVs are potent vaccines that can optimally prime immune responses against a broader range of SARS-CoV-2 variants. Thus, highly effective and safe LAVs are urgently needed to end the pandemic and help the world return to normalcy.

Cold adaptation is a process by which viruses gradually adapt to growth at suboptimal cold temperatures. Historically, it has been applied to generate attenuated respiratory RNA viruses, such as influenza virus, parainfluenza virus, respiratory syncytial virus, measles virus, rubella virus vaccine strains, and infectious bronchitis virus (a gammacoronavirus) [15,16,17,18,19,20,21]. Our goal was to generate cold-adapted and temperature-sensitive LAVs that exclusively/mainly replicate in the upper respiratory tract [12], which has a physiologically lower temperature than the lower respiratory tract (LRT), including the lungs. In this study, we generated such a SARS-CoV-2 mutant TS11 that replicated efficiently at lower temperature (32 °C), but not at 37–39 °C. Moreover, the pathogenicity and protective efficacy of the TS11 mutant was evaluated in Syrian hamsters. 

## 2. Materials and Methods

### 2.1. Viruses and Cells

Vero E6 cells (ATCC # C1008) were cultured in Dulbecco modified Eagle’s medium (DMEM) (Gibco, Carlsbad, CA, USA) supplemented with 10% heat-inactivated fetal bovine serum (FBS, Hyclone, Logan, UT, USA), and antibiotics/antimycotics (Gibco, Carlsbad, CA, USA). The SARS-CoV-2, USA-WA1/2020 (WA1) strain was obtained from BEI Resources. The virus was propagated once in Vero E6 cells at 37 °C to generate a WA1-P1 stock that was used in this study. Compared with the published WA1 sequence (GenBack accession No. MN985325), WA1-P1 contains one conservative mutation H245R in the S protein. Temperature-sensitive TS11 mutant (GenBack accession No. OP902892) was generated in this study. A D614G SARS-CoV-2 strain 14B (GenBack accession No. OK375077) was collected from an Ohio clinical sample in September 2020 and isolated in Vero E6 cells in our laboratory [22].

### 2.2. Cold Adaptation of SARS-CoV-2 WA1

The SARS-CoV-2 WA1 was serially passaged in Vero E6 cells in DMEM with 1% antibiotics/antimycotics (Gibco, Carlsbad, CA, USA) from 36 °C to 21 °C, with 1–2 passages at each temperature. Then, 5 additional passages were performed at 21 °C to further adapt the virus. Every passage of infected Vero E6 cells was frozen and thawed once, vortexed, and clarified by centrifugation.

### 2.3. Plaque Assay and Selection of the Temperature-Sensitive SARS-CoV-2 Clones

For plaque assay, monolayers of Vero E6 cells were inoculated with 10-fold serial dilutions of SARS-CoV-2 for 1 h. Then, the virus inoculum was removed, and the cell monolayers were washed three times with PBS. The cell monolayers were overlaid with 1.5% agarose (or 1% methylcellulose) in minimal essential medium (MEM) supplemented with 1% antibiotics/antimycotics, 1% HEPES, 1% non-essential amino acids and 2% heat-inactivated FBS. To clone the P34-21 °C-P5, the plaque assay was performed with 1.5% agarose overlay and individual plaques were picked up and cultured in Vero E6 cells at 32 °C for 4 days to produce a working virus stock. The above-selected virus clones were titrated using plaque assay at 32 °C for 4 days, and at 37 °C and 39 °C for 3 days. The diameter of 20 plaques was measured for each temperature/virus.

### 2.4. Multiple-Step Growth Kinetics of SARS-CoV-2 TS11

To evaluate the multi-step growth kinetics, Vero E6 cells were infected with a virus at a MOI of 0.01 for 1 h at 32 °C, and shifted and incubated at one of the respective temperatures 32 °C, 37 °C or 39 °C for 1 h, 24 h, 48 h, 72 h and 96 h. Then, the infected cells and supernatants were frozen and thawed once before harvesting. Then, the samples were vortexed, centrifuged, and the supernatants were tested for infectious titers on Vero E6 cells by plaque assay (overlayed with 1% methylcellulose) at 32 °C for 4 days. 

### 2.5. RNA Extraction and Reverse Transcription-PCR

Total cellular RNA was extracted using Trizol reagent (Invitrogen, Carlsbad, CA, USA) following the manufacturer’s instructions. We used the E and RNA-dependent-RNA polymerase (RdRp) gene-specific reverse transcription (RT)-qPCR assays to investigate the virus replication efficiency and to determine genomic equivalent titers, respectively. The positive-sense (+) RNA of SARS-CoV-2 was reverse transcribed using the E gene-specific reverse primer and SuperScript^®^ IV Reverse Transcriptase (Thermo Fisher, Waltham, MA, USA). The cDNA was subjected to quantitative PCR using SYBR green PCR mix (Life Technologies, Carlsbad, CA, USA) according to the manufacturer’s instructions. The subgenomic (sg) (+) E RNA was amplified with forward primer 5′-CGATCTCTTGTAGATCTGTTCTC-3′ and reverse primer 5′-ATATTGCAGCAGTACGCACACA-3′ [23]. The β-actin gene of Vero E6 cells was an internal control and amplified with forward primer 5′-AGGCTCTCTTCCAACCTTCCTT-3′ and reverse primer 5′-CGTACAGGTCTTTACGGATGTCCA-3′ [24]. Relative quantification is presented as fold changes relative to the control using the 2^ΔΔCT^ threshold method. TaqMan real-time RT-PCR (RT-qPCR) targeting the SARS-CoV-2 RdRp gene was performed using OneStep RT-PCR Kit (QIAGEN, Valencia, CA, USA) with primers and probe described previously [25]. To differentiate WA1 and TS11, conventional one-step RT-PCR was performed with primers (forward primer 5′- CTGACGGCGTAAAACACGTCTATCAGTTAC-3′ and reverse primer 5′- CTCCATTCTGGTTACTGCCAGTTGAATCTGAG-3′) with an annealing temperature of 55 °C. This pair of primers were designed in this study and covered the 371-nt-del in the ORF7b-ORF8 genes of SARS-CoV-2 and the product sizes for WA1 and TS11 were 769 bp and 398 bp, respectively. We used the OneStep RT-PCR Kit (QIAGEN) for this assay.

### 2.6. Next Generation Sequencing (NGS) and Sanger Sequencing

SARS-CoV-2 was sequenced by NGS as published [26]. Previously extracted RNA underwent 1st- and 2nd-strand cDNA synthesis (NEBNext Ultra II Non-Directional RNA Second Strand Synthesis Module; NEB, Ipswich, MA, USA), followed by sequencing using two different clinically validated amplicon-based methods. Some samples were analyzed using the SARS-CoV-2 Research Panel primers (ThermoFisher, Waltham, MA, USA) on the Ion Chef-S5 sequencer (ThermoFisher, Waltham, MA, USA) per the manufacturer’s conditions. Cultured virus and other samples were analyzed using the COVIDSeq kit (Illumina, San Diego, CA, USA) per the manufacturer’s conditions and sequenced on the NextSeq 550 sequencer (Illumina, San Diego, CA, USA). Strain typing by the two different assays was cross-validated using a set of 20 SARS-CoV-2-positive samples and 5 SARS-CoV-2-negative samples. Analysis tools include custom pipelines utilizing GATK and Mutect2 (Broad Institute) to determine variant percentages, and Dragen SARS-COVID variant detection (Illumina, San Diego, CA, USA). Viral sequences were strain-typed using NextStrain criteria (https://clades.nextstrain.org (accessed on 17 October 2022)). Pango lineages were determined according to https://pangolin.cog-uk.io (accessed on 23 April 2021) [27]. Sanger sequencing with SARS-CoV-2-specific primers was performed to confirm vague sequence regions generated by NGS.

### 2.7. Experimental Infection of Syrian Hamsters with SARS-CoV-2

All SARS-CoV-2 hamster studies were approved by the Ohio State University Institutional Animal Care and Use Committee. Syrian hamsters were purchased from Charles River (Wilmington, MA, USA). We conducted 2 experimental trials.

In the 1st trial, we tested whether the TS11 could cause clinical disease in hamsters. Eighteen 5–6-week-old male hamsters were randomly assigned to the SARS-CoV-2 WA1 group (n = 9, 3 hamster/cage) and the SARS-CoV-2 TS11 group (n = 9, 3 hamsters/cage). WA1 and TS11 groups were housed in the same animal room in the BSL3 facility, mimicking the natural conditions of vaccinated individuals who are subject to SARS-CoV-2 exposure at variable time points post-vaccination. The hamsters were anesthetized in the same box and sampled in the same biosafety cabinet (BSC) in the sequence of the TS11 group first followed by the WA1 group after cleaning the BSC using CaviCide spray (Metrex, Orange, CA, USA), waiting for 5 min, and changing gloves between handling the two groups of hamsters. The hamsters were inoculated intranasally (IN) with 4 × 10^4^ plaque-forming units (PFU) (in 60 µL) of WA1 or TS11. The hamsters were monitored for clinical signs daily. Their body weights and ear temperatures (in °F; transferred to °C) were measured, and nasal washes were collected before viral inoculation, daily for 1–6 days post-inoculation (dpi) and every other days during 7–13 dpi. Three hamsters in the same cage of each group were euthanized on 3 dpi, 6 dpi and 13 dpi. During necropsy, nasal turbinates, trachea, lungs, bronchoalveolar lavage fluid (BALF), and small intestinal tissues were harvested and frozen at −80 °C for the detection of infectious viral titers in each tissue. 

As TS11-inoculated hamsters were subsequently infected with WA1, a second experiment was carried out following decontamination. To remove environmental viruses, BSL3-Ag and necropsy spaces, which were used to house and handle the hamsters, were cleaned, sanitized and decontaminated using chlorine dioxide gas. No projects were run following the decontamination and prior to our trial. During this hamster study, no participating personnel worked with the WA1 strain. The animal cages were emptied, autoclaved and washed prior to reuse. 

In this second trial, we tested the pathogenicity and protective efficacy of TS11 against the challenge with a virulent SARS-CoV-2 D614G strain 14B in Syrian hamsters. Thirty 7–8 week-old male hamsters were randomly assigned to mock-challenge (Mock-C) and TS11 immunization-challenge (TS11-C) groups and immunized intranasally with culture medium and TS11 (8 × 10^4^ PFU per hamster), respectively. At 21 dpi, both groups were challenged intranasally with 14B (3 × 10^5^ TCID_50_ per hamster). Hamsters were observed for clinical signs daily. The body weights were measured and nasal washes were collected daily during the first four days after inoculation and every two or three days thereafter [from 5–20 dpi/5–12 days post-challenge (dpc)]. At 2 dpi, 6 dpi, 20 dpi, 23 dpi/2 dpc and 33 dpi/12 dpc, 3 hamsters/cage/group were euthanized to measure virus loads and conduct the histopathological examination. During necropsy, URT wash samples were collected by flushing the UTR from the upper trachea with 0.5 mL of culture medium and collected the effluents from the nostrils. Nasal turbinates, trachea, lungs and BALF (from the right lung), and testicles were harvested and frozen at −80 °C for the detection of infectious viral titers in each tissue. The left lungs were fixed in formalin solution for histopathological examination. Blood samples to measure viral neutralizing antibody titers were collected before vaccination and at euthanasia.

### 2.8. Histopathological Evaluation and Immunohistochemistry (IHC) for the Detection of SARS-CoV-2 Antigen

The left lung was fixed in 10% neutral formalin. Tissues were embedded, sectioned (3.5 µm), and stained with Gill’s hematoxylin and eosin (H&E) for light microscopic examination as described previously [28]. The Mock-C hamsters at 2 dpi or 12 dpc, histopathologic lesions mainly consisted of (1) accumulation of necrotic cells and inflammatory cells in alveolar, or bronchial or bronchiolar lumens; (2) thickening of alveolar septa by type 2 pneumocyte hyperplasia and inflammatory cell infiltration; and (3) vascular abnormalities (Supplemental Figure S1) Vascular lesions included (1) the presence of hypertrophied endothelial cells, (2) the aggregates of inflammatory cells beneath or within the endothelial cell layer, or within the vascular wall, (3) the hypereosinophilia and degeneration of the tunica media, or (4) perivascular edema and/or lymphocytic cuffing (Supplemental Figure S1), consistent with a previous report [29]. The major histopathologic lesions were used as parameters to evaluate the severity of pneumonia, as described previously with slight modifications [30]. Briefly, the severity of alveolitis and bronchitis/bronchiolitis or thickening of alveolar septa was evaluated based on the percentage of the affected area in each microscopic area (×25) of a pulmonary section, as follows: 0, no lesions; 1, affected area ≤10%; 2, affected area 10–50%; and 3, affected area ≥50%. However, the severity of vascular lesions was evaluated, as follows: 0, no lesions; 1, focal or multifocal, mild lesions; 2, multifocal, moderate lesions; and 3, multifocal, severe lesions. For each parameter, mean values of all scores from three different areas in each cranial, middle, and caudal region of the left lung were calculated and then cumulated (ranging from 0 to 9).

The formalin-fixed lung tissue sections were tested by IHC for the detection of SARS-CoV-2 antigen, as previously described with slight modifications [31]. A recombinant monoclonal antibody against SARS-CoV-2 N protein (Kerafast, Boston, MA, USA) was used as the primary antibody and a non-biotin polymerized horseradish peroxidase system (BioGenex Laboratories, San Ramon, CA, USA) was used for visualization as brown staining. Stained tissues were counterstained with hematoxylin. SARS-CoV-2 antigen-positive IHC scores were computed by estimating the intensity and frequency of IHC-positive cells in each given microscopic area (×100) of a pulmonary section based on the following criteria: 0, no positive cells; 1, low numbers of positive cells showed focal or multifocal, mild staining; 2, moderate numbers of positive cells showed multifocal or multifocal to coalescing, moderate staining; and 3, high numbers of positive cells showed diffuse strong staining. Mean values of all scores from three different areas in each cranial, middle, and caudal region of the left lung were calculated (ranging from 0 to 3).

### 2.9. Measurement of Infectious Viral Titers and Serum Viral Neutralizing (VN) Antibody Titers

Hamster tissues were weighed and homogenized for 30 s at 4.0 m/s, using the TissueLyser II (Qiagen) in 500 µL DMEM containing 2% FBS and 1% Antibiotic-Antimycotic. The nasal washes, tissue homogenates, URT washes and BALF samples were clarified by centrifugation at ~ 2000× *g* for 5 min and the supernatants were collected for measurement of infectious virus titers in median tissue culture infectious dose 50 (TCID_50_) using 96-well plates. Briefly, Vero E6 cells were seeded into 96-well plates one day before the testing. The cell monolayers were washed twice before inoculation (100 µL of 10-fold serial dilutions of samples per well and four replicates per dilution). The plates were incubated at 32 °C for the TS11 mutant or at 37 °C for the 14B strain. Viral cytopathic effects (CPEs) were observed at 3 dpi and virus titers were calculated by the Reed-Muench method [32].

Serum VN antibody titers were measured by TCID_50_-reduction neutralization assay. To prepare the serum-virus mixture, 4-fold serially diluted serum samples were mixed with an equal volume of SARS-CoV-2 14B strain (100 TCID_50_ of virus/well for the final inoculation). The serum-virus mixtures were incubated at 37 °C for 1 hr before inoculation of the Vero E6 cell monolayers, with four replicates per dilution. Virus control, medium control, and positive and negative serum controls were included. The plates were incubated at 37 °C for 3 days. Viral CPEs were observed and the absence of CPEs indicates that the virus was neutralized. All serum samples were tested at the same time. VN antibody titers were calculated by the Reed-Muench method [32].

### 2.10. Statistical Analysis

The statistical analyses were performed using GraphPad Prism, version 8. The comparison of values of TS11 and WA1 were analyzed by Student’s *t*-test. RNA and infectious viral titers were analyzed by one-way ANOVA followed by Dennett’s test. A *p* value of less than 0.05 was considered significantly different.

## 3. Results

### 3.1. Generation of a Cold-Adapted and Temperature-Sensitive SARS-CoV-2 TS11

**Figure 1:**
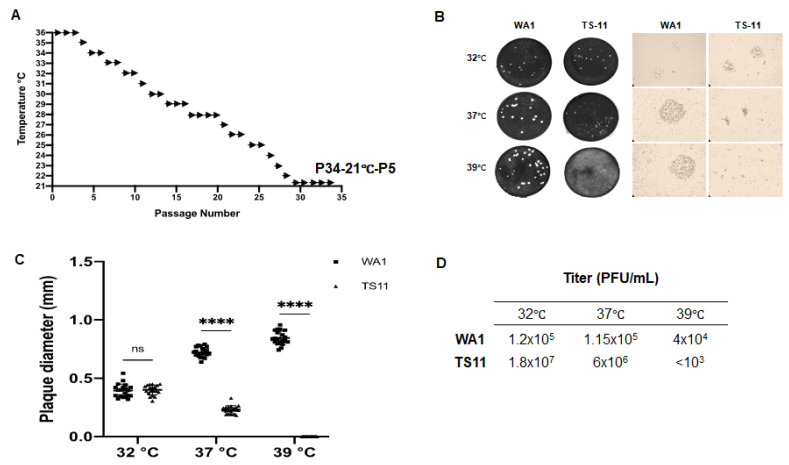
Generation of a cold-adapted/temperature-sensitive SARS-CoV-2-WA1 mutant (TS11). (**A**) Overview of the passaging history that generated the cold-adapted TS11 strain from SARS-CoV-2 WA1. (**B**) Representative images of the viral plaques formed by WA1 and TS11 cultured at different temperatures (32 °C, 37 °C and 39 °C): left panel shows plaque appearance by eye, and the right panel shows the plaques observed under a light microscope. (**C**) The diameter of plaques of WA1 and TS11 cultured at 32 °C, 37 °C and 39 °C. The diameter means of 20 plaques of TS11 and WA1 viruses were compared by Student’s t test (****, *p* < 0.001; ns, not significant). (**D**) The efficiency of plating (or plaque forming efficiency) of WA1 and TS11 virus stock at 32 °C, 37 °C and 39 °C.

To obtain TS SARS-CoV-2 variants that can replicate efficiently within the temperature range observed in the URT (25–33 °C) but not in the LRT (37–39 °C), the SARS-CoV-2 WA1 strain was stepwise passaged in Vero E6 cells using temperatures from 36 °C to 21 °C, with 1–5 passages at each temperature, for a total of 34 passages (Figure 1A). The virus was designated as P34-21-P5 and was further cloned by plaque assay. Initially, 52 plaques were picked and individual plaques of virus were cultured at 32 °C to produce a working virus stock. Clone #11 showed the desired cold-adaptation and temperature-sensitive phenotype and was designated as TS11 mutant. The plaques of TS11 were much smaller than those of the WA1 strain at 37 °C and 39 °C (Figure 1B,C). TS11 formed significantly smaller plaques at 37 °C or 39 °C than at 32 °C, whereas WA1 formed larger plaques at 37 °C or 39 °C than at 32 °C. Additionally, the infectious titers of TS11 were <10^3^ PFU/mL at 39 °C but 1.8 × 10^7^ PFU/mL at 32 °C. The efficiency of plating (EOP) is defined as the ratio of the viral titer at 39 °C or 37 °C to the viral titer at 32 °C; Viruses that have an EOP ≤0.01 were defined as TS mutants [18]. The EOP of TS11 was <0.00006. In contrast, WA1 had similar infectious titers at 32 °C, 37 °C and 39 °C (Figure 1D). These results demonstrated that SARS-CoV-2 TS11 is a cold-adapted TS SARS-CoV-2 mutant in culture.

### 3.2. Characterization of the SARS-CoV-2 TS11 Mutant

**Figure 2:**
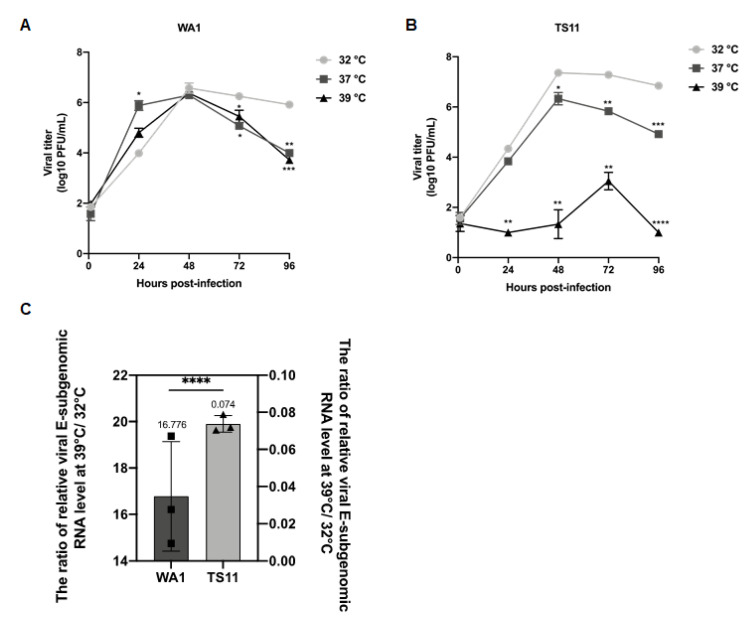
Characterization of the SARS-CoV-2 TS11 in Vero E6 cells. Multi-step growth kinetics of SARS-CoV-2 WA1 (**A**) and SARS-CoV-2 TS11 (**B**) at 32 °C, 37 °C and 39 °C. Vero E6 cells were inoculated with SARS-CoV-2 WA1 and SARS-CoV-2 TS11 at a MOI of 0.01 at 32 °C, 37 °C or 39 °C. Infected cells were harvested at the indicated time points, and virus titers were determined by plaque assays. The ratio of relative viral E subgenomic (+) RNA level at 39 °C:32 °C (**C**). Vero E6 cells were infected with SARS-CoV-2 WA1 or TS11 at an MOI of 1.0 at 32 °C for 1 h. After removing the virus inoculum and washing cells, the plates were cultured at 32 °C or 39 °C for an additional 7 h. Total RNA was extracted from the infected cells, and the relative levels of SARS-CoV-2 E gene subgenomic (+) RNA were determined by reverse transcription followed by quantitative PCR using actin gene as an internal control. The data are presented as mean ± standard deviation of triplicates. At each time point, data were analyzed by one-way ANOVA followed by Dennett’s test by comparing data of each group with values at 32 °C (*, *p* < 0.05; **, *p* < 0.01; ***, *p* < 0.005; ****, *p* < 0.001).

SARS-CoV-2 TS11 was subcloned and one plaque was selected for the generation of a virus pool for further in vitro characterization. Multi-step growth kinetics experiments were performed in Vero E6 cells infected with SARS-CoV-2 WA1 or TS11 at a multiplicity of infection (MOI) of 0.01 and incubated at 32 °C, 37 °C and 39 °C, respectively. Virus titers were measured at 1, 24, 48, 72, and 96 h post-inoculation (hpi) by plaque assay. The parental SARS-CoV-2 WA1 grew efficiently in Vero E6 cells and reached similar peak titers at 48 hpi at three temperatures (Figure 2A). In contrast, TS11 replicated slowly and reached peak titers later at 39 °C (at 72 hpi) than at 32 °C and 37 °C (at 48 hpi) and the peak titers at 37 °C and 39 °C were significantly lower than that at 32 °C, suggesting that the replication of TS11 was severely restricted at 39 °C.

To further investigate the impaired replication of TS11, we assessed the level of virus production at several temperatures. Vero E6 cells were inoculated with TS11 or WA1 at a MOI of 1 and incubated at 32 °C for 1 h, then the culture temperature was shifted to 39 °C or maintained at 32 °C for additional 7 h, as described previously [33]. The supernatant was removed, and the cells were lysed using TRIzol. The relative levels of SARS-CoV-2 subgenomic (+) E RNA were tested by reverse transcription followed by SYBR green real-time PCR using actin as an internal control. The ratio of WA1 subgenomic (+) E RNA levels at 39 °C to 32 °C was 16.776, whereas the ratio of TS11 was 0.074 (Figure 2C), indicating that the RNA replication of WA1 at 39 °C was much faster than at 32 °C. In contrast, the replication of TS11 was extremely restricted at 39 °C.

### 3.3. Genomic Analysis of the SARS-CoV-2 TS11

The complete genome of the TS11 virus stock was sequenced using full SARS-CoV-2 genome next generation sequencing (NGS) followed by Sanger sequencing confirmation. Nucleotide and amino acid changes of SARS-CoV-2 TS11 compared to the parental (SARS-CoV-2 WA1) strain are listed in Table 1. There were two deletions in TS11: (1) a 12-amino acid-deletion located in the junction of S1 and S2 region including the furin cleavage site (PRRAR); and (2) a 371-nucleotide-deletion resulting in partial orf7b (1–17 amino acid residues) and the complete deletion of the orf8 protein. In addition, non-conservative mutations were found in nsp3 (M494K, A579V, T763M, N793S, T1456I), nsp16 (H69Y), S (S813I), E (A32V), orf7a (S44L), and N (T198I) proteins.

### 3.4. SARS-CoV-2 TS11 Mutant Did Not Cause Clinical Disease in Syrian Hamsters Even following Co-Infection with WA1 Strain

**Figure 3:**
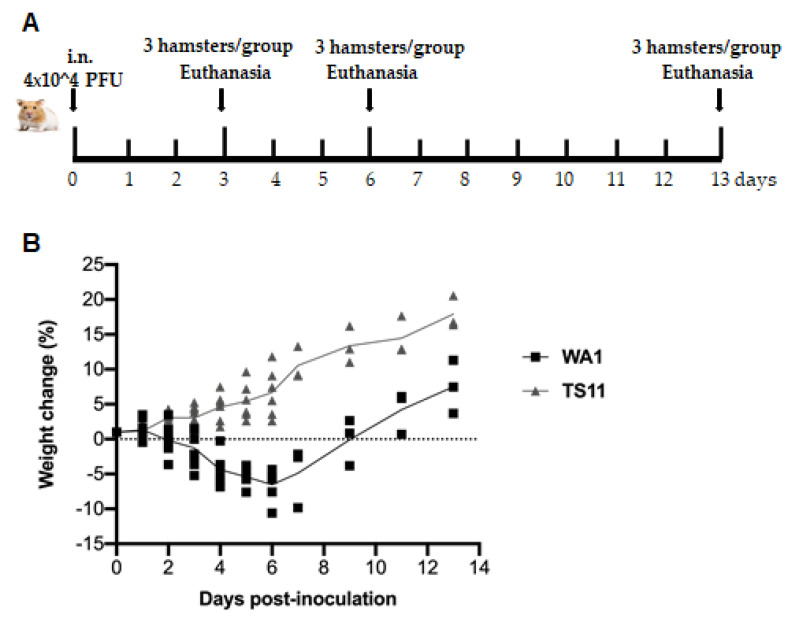

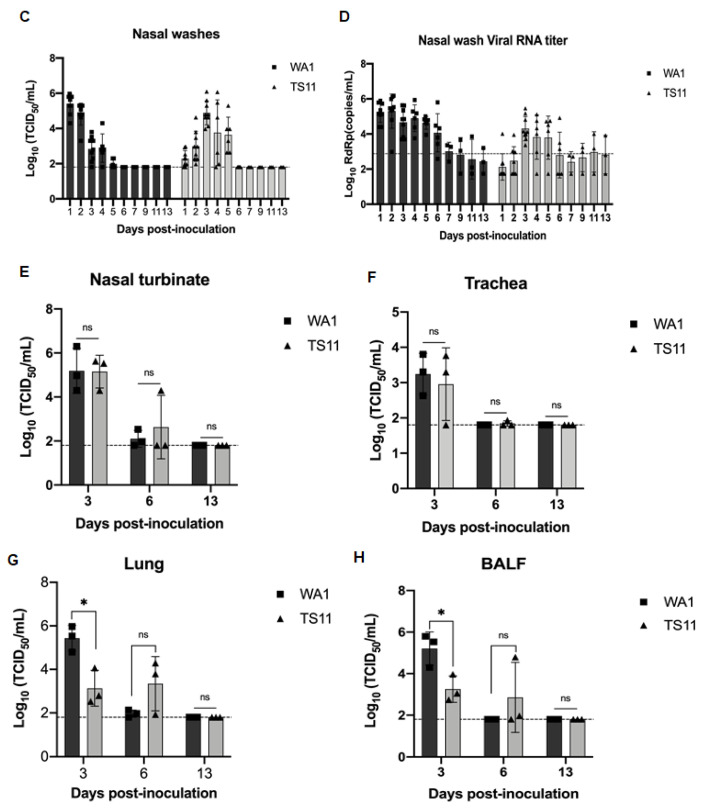
SARS-CoV-2 TS11 did not cause disease in Syrian hamsters even during subsequent co-infection with WA1 strain. (**A**) Schematic procedure for the Syrian hamster study. Two groups of hamsters (n = 9 per group) were intranasally (i.n.) inoculated with 4 × 10^4^ PFU/hamster of SARS-CoV-2 WA1 or SARS-CoV-2 TS11. (**B**) Body weights were measured daily from 1–7 dpi followed by every two days. Nasal washes were collected daily from 1–7 dpi followed by every two days and infectious viral titers (**C**) and viral RNA titers (**D**) were determined by TCID_50_ assay and RT-qPCR targeting the RdRp gene, respectively. At 3 dpi, 6 dpi, and 13 dpi, 3 hamsters per group were euthanized and the infectious virus titers in the nasal turbinate (**E**), trachea (**F**), lung (**G**), and BALF (**H**) samples were assessed by the TCID_50_ assay. The dashed lines in C-H indicate the detection limit. The data were presented as mean ± standard deviations. At each time point, values of the TS11 and WA1 groups were compared by Student’s t test (*, *p* < 0.05; ns, not significant).

**Figure 4:**
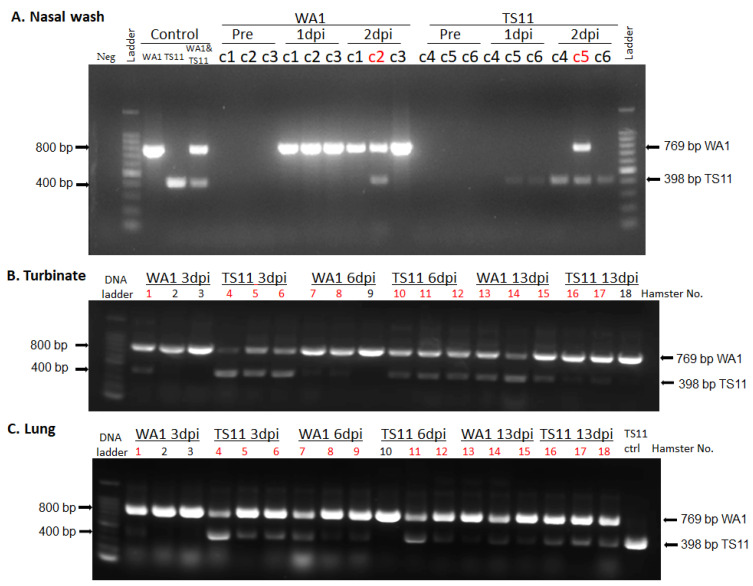
The detection of TS11 and WA1 viral RNA in the nasal wash (**A**), nasal turbinate (**B**) and lung (**C**) samples of hamsters using the RT-PCR that differentiates between WA1 (769 bp) and TS11 (398 bp). WA1 RNA, TS11 RNA and the 1:1 mixture of WA1 and TS11 RNA based on infectious titers (WA1&TS11) were used as positive controls; water was used as a negative (Neg) control. C1–C6 indicate randomly selected one of the three hamsters in cage #1–cage #6. The red text indicate that the sample was positive for both TS11 and WA1.

Using the Syrian Hamster model to assess pathogenicity, replication and tissue tropism (Figure 3A), we found that animals infected with WA1 lost body weight starting from 2 dpi to 8 dpi with a peak weight loss at 6 dpi. Then, the animals started gaining body weight gradually, which was restored to the initial body weight level by 9 dpi and kept increasing thereafter (Figure 3B). In contrast, animals infected with TS11 gradually gained body weight from 1 dpi to 13 dpi and did not show any clinical signs. The amount of infectious virus shed in nasal washes in the WA1 group peaked at 1 dpi, whereas hamsters in the TS11 group showed peak titers later at 3 dpi (Figure 3C). Both groups of hamsters had no detectable infectious virus in their nasal washes at 6 dpi. The viral RNA shedding titers in nasal washes (Figure 3D) showed a similar trend to the infectious viral titers. Tissue samples from the lungs, nasal turbinates, tracheas, small intestines and BALF samples were collected and analyzed for infectious viral titers at 3 dpi, 6 dpi, and 13 dpi (Figure 3E–H). The titers in the nasal turbinate and trachea samples were similar between the two groups at 3 dpi and 6 dpi. Hamsters in the TS11 group had significantly lower titers (~2 Log_10_-lower) in the lungs and BALFs than hamsters in the WA1 group at 3 dpi but showed similar low titers at 6 dpi. Either no detectable or extremely low levels of infectious virus was detected in both groups at 13 dpi. No infectious virus was detected in the small intestines of the experimental hamsters (data not shown). No significant body temperature changes were observed between pre-inoculation and post-inoculation and the hamsters’ body temperature was 36.3 ± 0.2 °C during the experiment. However, we found that the two groups of hamsters were co-infected with WA1 and TS11 starting from 2 dpi using an RT-PCR assay that differentiates between WA1 and TS11 viruses (Figure 4).

### 3.5. TS11 Was Attenuated in Syrian Hamsters and Induced Protection against Clinical Disease following Challenge with a Heterologous SARS-CoV-2

**Figure 5:**
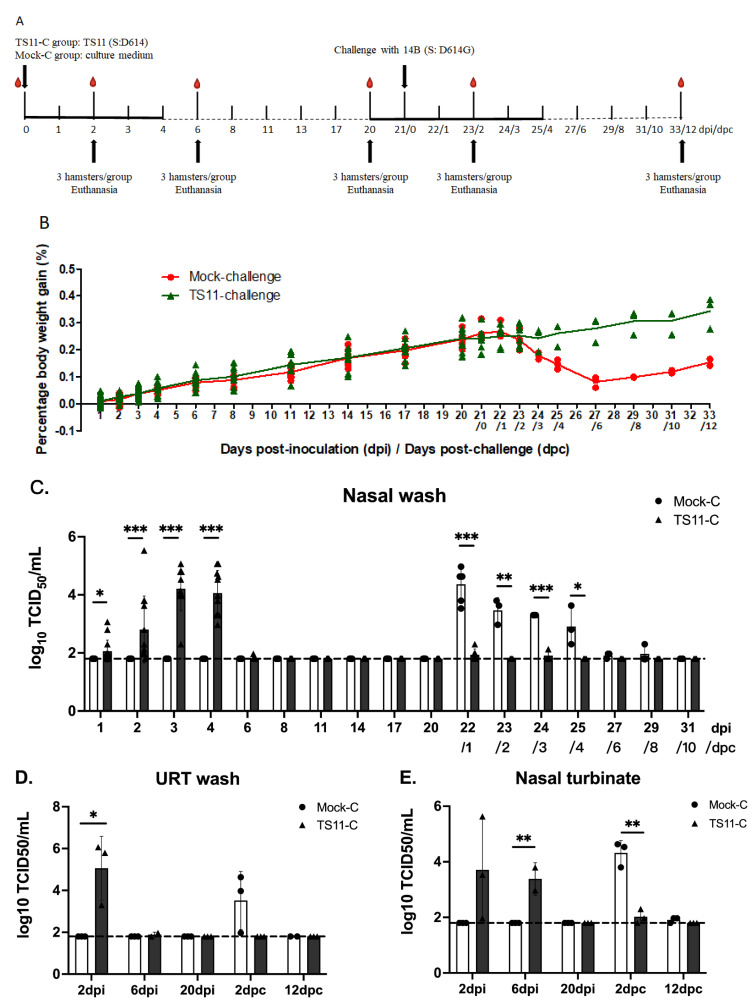

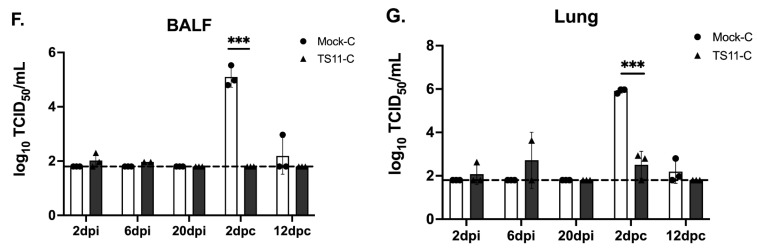

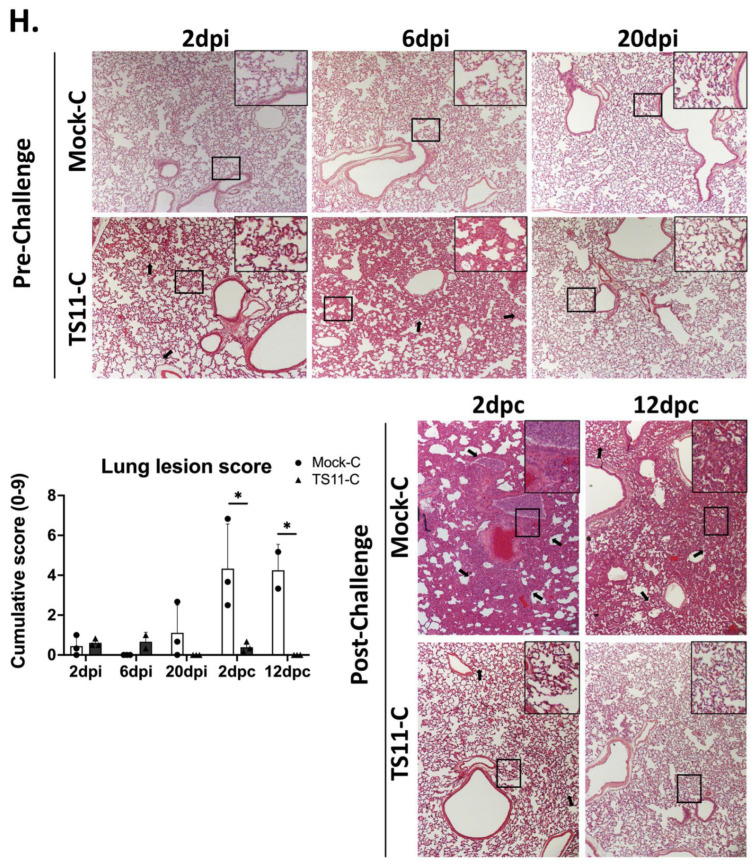

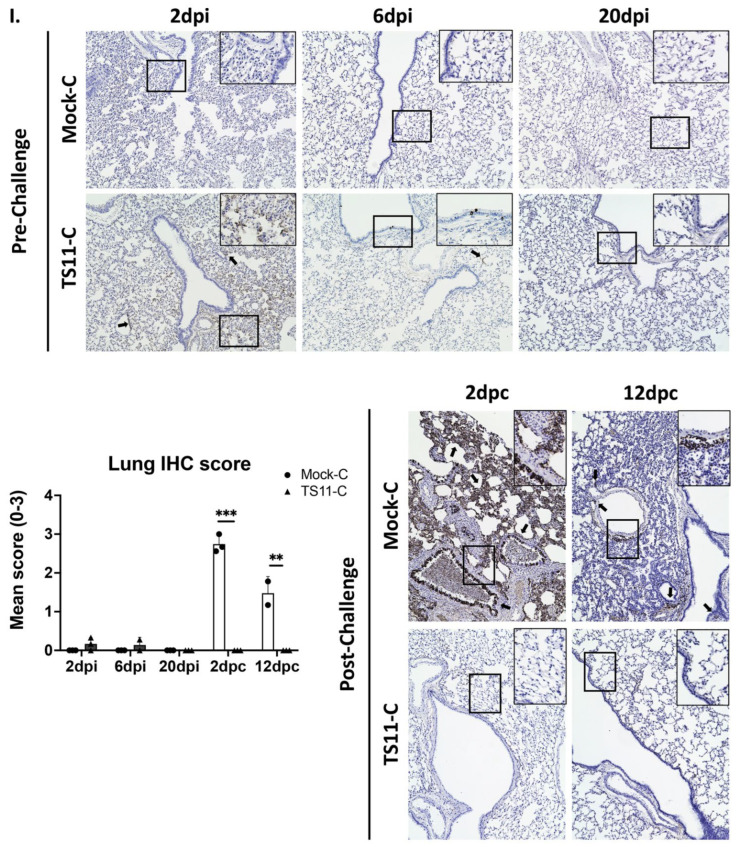

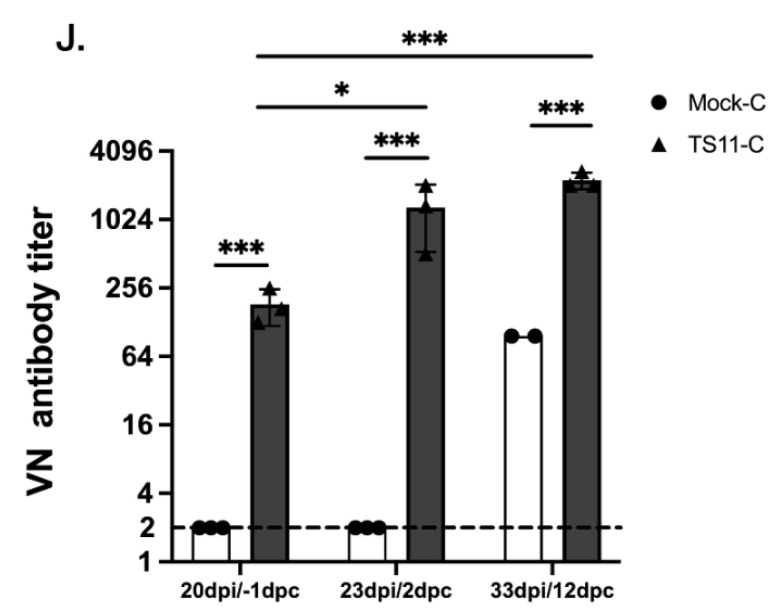
SARS-CoV-2 TS11 was attenuated in hamsters and conferred complete protection post challenge with a virulent heterologous D614G strain 14B. (**A**) Schematic procedure for the Syrian hamster study. Two groups of hamsters (n = 15 per group) were intranasally (i.n.) inoculated with 8 × 10^4^ PFU/hamster of SARS-CoV-2 TS11 (for TS11-Challenge group) or culture medium (for Mock-challenge group). At 21 days post-inoculation (dpi), both groups of hamsters were challenged i.n. with a virulent heterologous D614G strain 14B (3 × 10^5^ TCID_50_ per hamster). (**B**) Body weights were measured daily from 1–4 dpi or days post challenge (dpc) followed by every 2–3 days. (**C**) Nasal washes were collected daily from 1–4 dpi/dpc followed by every 2–3 days and infectious viral titers were determined using Vero E6 cells. At 2 dpi, 6 dpi, 20 dpi, 23 dpi/2 dpc, and 33 dpi/12 dpc, 3 hamsters per group were euthanized. The infectious virus titers in the upper respiratory tract (URT) wash (**D**), nasal turbinates (**E**), Bronchoalveolar lavage fluid (BALF) (**F**), and lungs (**G**) were assessed by the TCID_50_ assay. The dashed lines in C g indicate the detection limit. The histopathological changes of lungs were examined after hematoxylin and eosin staining (Arrows indicate lesions) (**H**) and immunohistochemical (IHC) staining of SARS-CoV-2 N proteins (brown color) (**I**). Black arrows indicated lesions or positive signals. Original magnification, ×60 (panels in H) or ×100 (panels in I). A typical area was also shown in an amplified box in the top right corner. Serum viral neutralizing (VN) antibody titers were tested using TCID_50_-reduction assay (**J**). The data were presented as mean ± standard deviation (SD). At each time point, values of the TS11 and WA1 groups were compared by Student’s t test (*, *p* < 0.05; **, *p* < 0.01; ***, *p* < 0.005).

To confirm the attenuated phenotype of TS11 in a single virus infection experiment, we performed a second hamster study (Figure 5A) and a heterologous challenge study. Similar to the mock group, TS11-vaccinated hamsters did not show any clinical signs and continuously gained body weight (Figure 5B). There was no infectious virus shedding in the mock group. Nasal virus shedding started at 1 dpi, peaked at 3 dpi, and subsided by 6 dpi (Figure 5C). TS11 replicated to higher titers in the nasal cavity at 2 dpi than at 6 dpi (Figure 5D,E). However, the titers were higher in the lungs at 6 dpi vs. 2 dpi (Figure 5F,G). TS11 infection caused very mild alveolitis in the lungs with low amounts of viral N protein detected from the lungs (Figure 5H,I). No infectious virus was detected in the testicles (data not shown). At 20 dpi, no virus or inflammation was detected in any tissues (Figure 5C,I), indicating that the hamsters cleared the infection prior to the challenge. These results indicate that the TS11 mutant mainly replicated in the URT and was attenuated in Syrian hamsters.

After challenge with the heterologous virulent D614G strain 14B, hamsters in the Mock-C group progressively lost body weight starting from 1 dpc with the weight loss peaking at 27 dpi/6 dpc (Figure 5B). In contrast, the animals in the TS11-C group gained body weight continuously. Infectious 14B virus was shed highest at 1 dpc in the Mock-C group and gradually decreased to the levels below the detection limit at 6 dpc (Figure 5C). In contrast, significantly lower virus titers were detected in the TS11-C group post-challenge. Similar results were observed in the tissues collected from the URT (nasal washes and nasal turbinate homogenates) and LRT (lung homogenates and BALF samples) at 2 dpc (Figure 5D,G). 

At 2 dpc, histopathological examination demonstrated that the lungs of the Mock-C group hamsters developed moderate to severe alveolitis characterized by necrosis of alveolar epithelial cells, and severely occluded bronchi or bronchioles by accumulation of necrotic cells and inflammatory cells, such as neutrophils, in the lumens (Figure 5H). In 1 of 3 and 2 of 2 Mock-C group hamsters at 2 dpc and 12 dpc, respectively, there was also mild to moderate thickening of alveolar septa caused by type 2 pneumocyte hyperplasia and inflammatory cell infiltration. However, TS11-C group hamsters had only mild and no pulmonary lesions at 2 dpc and 12 dpc, respectively (Figure 5H). By IHC, large and moderate amounts of SARS-CoV-2 N antigens were present in the lungs of Mock-C group hamsters at 2 dpc and 12 dpc, respectively (Figure 5I). In contrast, there was no N protein staining in the lungs of TS11-C group at 2 dpc and 12 dpc. At 12 dpc, lesions in the lungs of the TS11-C hamsters disappeared; however, pronounced lesions and viral antigens were still present in the lungs of Mock-C hamsters.

We tested the VN antibody titers to the challenge virus 14B in the serum samples collected before (at 20 dpi) and after challenge (at 23 dpi/2 dpc and 33 dpi/12 dpc). TS11 infection induced an intermediate level of VN antibody titers (177) in the TS11-C at 20 dpi, and the challenge boosted the VN antibody titers to a high level (1123 and 2246 at 2 dpic and 12 dpc, respectively) (Figure 5J). These results correlated with the protection levels observed in this group of hamsters post-challenge. The virulent 14B infection induced intermediate level of VN antibody titers (97) in the Mock-C hamsters at 12 dpc. In summary, TS11 mutant was attenuated and induced protection after the heterologous SARS-CoV-2 challenge in Syrian hamsters.

There was one outlier hamster [20] in the TS11-C group. It shed high titers of infectious virus in the lung homogenate (5.6 log_10_ TCID_50_/mL) and the BALF sample (5.3 log_10_ TCID_50_/mL) at 6 dpi. The hamster was gaining body weight from 1–3 dpi but lost 4% body weight from 4 dpi (141.9 g) to 6 dpi (136.3 g). Differentiation RT-PCR was performed and sequence analyses of the amplicons showed that the virus-specific 707 bp-fragment shared 100% nucleotide identity with the WA1 strain. Then, we sequenced the genomes of the viruses detected in the nasal wash and lung homogenate samples of H#20 collected at 6 dpi by NGS. These two viral genomes contained intact orf7b and orf8 as WA1 but shared high sequence identity with TS11 in the rest of the genome except for an additional deletion in the N protein (N:S193-). We designated this virus as a TS11-B variant and its genome was similar to the genomes of the other two clones TS15 and TS28 from P34-21-P5. As we did not use WA1 in the second hamster experiment and TS11 was derived from WA1, TS11-B is probably an intermediate virus from WA1 to TS11 and the inoculum contained TS11 and TS11-B as the predominant and minor populations. Further analyses of the nasal wash samples collected at 1–4 dpi from H#20 and the other two hamsters [19 and H#21] housed in the same cage revealed that the TS11-B variant was not detected from the nasal wash samples until 4 dpi, and was not detected from the samples of H#19 and H#21. Therefore, H#20 was co-infected with attenuated TS11 and virulent TS11-B and its data between 4 and 6 dpi were excluded from Figure 5.

## 4. Discussion

Here, we present our results on the development and characterization of a cold-adapted temperature-sensitive variant of SARS-CoV-2 (TS11) that shows attenuated phenotype both in vitro and in Syrian hamsters. Compared with its parent virus, TS11 has sequence alterations in the furin cleavage site of S and other genes including nsp3 (Table 1). The nsp3 of SARS-CoV-2 is the largest viral protein and is an essential component of the replication/transcription complex [34]. The nsp3 SARS-Unique Domain (SUD) includes three distinct subdomains: macrodomain II (Mac2), macrodomain III (Mac3), and domain preceding ubiquitin-like domain 2 (Ubl2) and papain-like protease 2 (PL2^pro^) (DPUP) [35]. Previously, Deng et al. [36] reported a temperature-sensitive mouse hepatitis virus (MHV), a betacoronavirus (tsNC11) whose phenotype was induced by the mutations in its nsp3 suggesting a similar mechanism for TS11 which showed changes in Mac2, Mac3 and DPUP. 

Mac2 is dispensable for the SARS-CoV replication/transcription complex, while Mac3 is necessary. The Mac3 domain of SARS-CoV nsp3 can interact with DNA or RNA G-quadruplexes (G4) and is essential for SARS-CoV replication [37]. It has been shown that Mac2 and Mac3 of SARS-CoV-2 also interact with G4 structures [38]. Recently, the nsp3 Mac2 of both SARS-CoV and SARS-CoV-2 have been shown to interact with human poly(A) binding protein interacting protein 1 (Paip-1), a component of the cellular translation machinery [39]. For SARS-CoV, this interaction between Mac2 and Paip1 stimulates viral RNA translation but does not affect the translation of cellular mRNAs. The M494K mutation of SARS-CoV-2 TS11 located in Mac2 changes the amino acid methionine with an S-methyl thioether side chain to lysine with a positively charged polar side chain. This M494K mutation may influence the interactions between Mac2 and Paip1, thereby influencing the viral RNA translation. The A579V mutation is in the Mac3 domain. 

The Ubl2 and PL2^pro^ domains are conserved in all CoVs. The exact functional role of the Ubl2 domain is not clear, while the PL2^pro^ possesses proteolytic, deubiquitinating, and deISGylating activities [35]. The T763M is a non-conservative mutation within the PL2^pro^ domain and might alter its functions. It appears that T1456I mutation in the nsp3 ectodomain (3Ecto) is not critical for the domain functionality since the conserved cysteines and the two glycosylation sites (Asn1431 and Asn1434) essential for 3Ecto functions were retained [35]. Whether some or all of these mutations in the nsp3 of SARS-CoV-2 TS11 mutant are essential to induce temperature-sensitivity remains to be determined using reverse genetics.

The furin cleavage site of the SARS-CoV-2 WA1 S protein plays a critical role in the efficient infection of the LRT [40]. A SARS-CoV-2 WA1 variant lacking the furin cleavage site had decreased replication in a human respiratory cell line, was attenuated in both hamsters and K18-hACE2 transgenic mice [41], and had reduced transmission in ferrets [42]. Therefore, the 12-amino acid deletion including the furin cleavage site may contribute to the attenuated phenotype of SARS-CoV-2 TS11 in hamsters. However, we observed that despite the attenuated phenotype, TS11 was still capable of efficient transmission to the WA1-inoculated hamsters starting at 2 dpi, which was similar to the transmission of WA1 to the TS11-inoculated hamsters. The orf8 of SARS-CoV-2 is a glycoprotein that is secreted as homodimers [43]. It can interact with the IL17 receptor A to modulate IL-17 signaling [44], decreasing IFN-β production [45] and downregulating the surface expression of MHC class I to evade immune responses [46].

**Figure 6:**
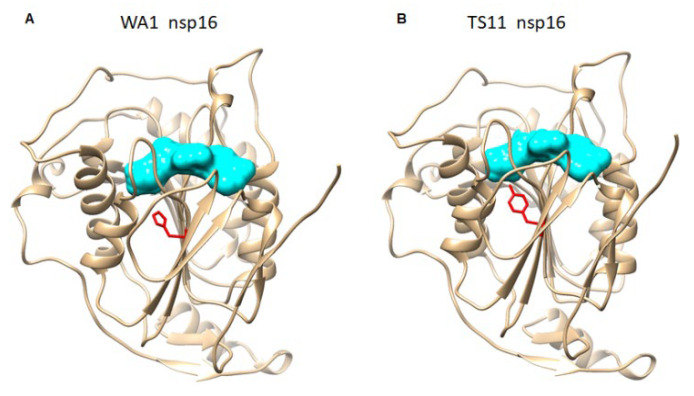
Three-dimensional structural analyses of the nsp16 proteins of SARS-CoV-2 WA1 (**A**) and SARS-CoV-2 TS11 (**B**). They were modeled based on the structure of nsp16 of SARS-CoV-2 WA1 from the Protein Data Bank (PDB accession number 6WKS). S-adenosyl methionine is in Cyan. Tyrosine (TYR); Histidine [18].

A SARS-CoV-2 variant carrying a 382-nucleotide-deletion in ORF8 was first detected in 23.6% (45/191) of samples in Singapore in January 2020 [47]. It exhibited dramatically higher replicative fitness in vitro than the wildtype SARS-CoV-2. This 382-nucleotide-deletion was also found to be associated with lower concentrations of inflammatory cytokines and a milder disease [48]. Subsequently, variable length of the ORF7b and/or ORF8 deletions in SARS-CoV-2 variants were found in patients in Taiwan [49], Bangladesh [50], Australia, and Spain [51]. While orf8 is a rapidly evolving accessory protein and is involved in immune responses, its expression is not essential for SARS-CoV-2 infection and transmission [52]. Therefore, the partial deletion of orf7b and the complete deletion of orf8 may contribute to the attenuated phenotype of SARS-CoV-2 TS11 in hamsters. The nsp16 of CoVs is a conserved SAM-dependent 2′-O-methyltransferas (2′-O-Mtase) to form a cap-1 structure, which is critical for the evasion of host innate immunity [53,54]. Although TS11 does not have mutations in the catalytic tetrad (K-D-K-E) of the enzyme, the 69H of nsp16 is near the S-Adenosyl methionine [55]-binding pocket [56] (Figure 6A). In TS11 mutant, the 69H with a positively charged side chain is replaced by tyrosine with a non-charged polar side group, which could reduce the SAM affinity of nsp16 (Figure 6B). Therefore, the H69Y substitution in TS11 nsp16 could reduce the enzyme activity, resulting in enhanced innate immune responses and attenuation. However, whether these mutations found in the TS11 lead to attenuation remains to be determined using a reverse genetics system.

Our hamster studies showed that SARS-CoV-2 TS11 is attenuated in these animals. The TS11-inoculated hamsters shed lower infectious viruses in the lungs and BALFs than the WA1-inoculated hamsters. Moreover, these TS11-inoculated hamsters did not show any weight loss during the entire infection phase, whereas WA1-inoculated hamsters had up to 6% weight loss. In addition, priming the hamsters’ immunity with a high dose of TS11 prevented the virulent WA1 disease during a mixed infection. In contrast, TS11 failed to alleviate the WA1-induced disease when the hamsters were first inoculated with a high dose of WA1. This protective effect of prior TS11 inoculation may be due to several possibilities that lead to the inhibition of WA1 replication: (1) As different groups of hamsters were housed in different cages, the TS11-inoculated hamsters probably were infected with a low dose of WA1 through the contaminated box used for anesthesia; (2) Prior TS11 infection generated a large amount of TS11 viruses that can interfere with the replication of subsequent low amount of WA1 in hamsters; and (3) since TS11 has a mutation in nsp16 and deletions spanning the orf7b and orf8 accessory proteins that can blunt innate immune responses, prior TS11 infection may induce strong and lasting host innate immune responses, allowing the host to control replication of subsequent WA1 exposure. On the other hand, when large amounts of WA1 infected the hamster first, virus production may effectively overcome the host innate immune responses rendering TS11 cross-protection less effective. These alternate hypotheses need to be addressed more directly in future studies. 

Nevertheless, considering that vaccinated humans can be exposed to and infected with wildtype SARS-CoV-2 in the real world, this property of TS11 is a favorable feature for a LAV. The attenuated phenotype of TS11 was further confirmed in the second hamster trial. Additionally, TS11 infection induced protective immunity, including the generation of moderate VN antibody titers, and efficiently protected the hamsters from disease following challenge with a high dose of virulent heterologous D614G strain 14B. Therefore, TS11 is a promising LAV candidate and deserves further evaluation and development.

In the second hamster study, the TS11-B variant was not generated by a recombination event during the co-infection with two parental strains (WA1 and TS11) because there was no WA1 virus after facility decontamination. Instead, the genome of TS11-B was similar to two clones TS15 and TS28, indicating that TS11-B is an intermediate between WA1 and TS11 during the cold-adaptation process. Recent studies indicate that viruses exist in the form of extracellular vesicle-cloaked viral clusters and free virus aggregates in addition to free single virions [57]. Although our TS11 virus stock was purified by two cycles of plaque assays and TS11-B was undetectable by the differentiation RT-PCR and NGS, it is possible that the TS11 inoculum contains TS11-B at an extremely low level. On the other hand, H#20 may be an outlier individual in which the extremely low amount of TS11-B outcompeted the dominant TS11 during replication resulting in the observed weight loss during the later stage (4–6 dpi) of acute infection. The host factors involved in this process necessitate future investigation. Our results also highlight the importance of using infectious clones to prepare large quantities of homogeneous virus inoculum. The limitation of this study is that SARS-CoV-2-infected Syrian hamsters do not develop fever, so we could not assess whether TS11 replicates in the lungs when the animal has a fever, which can be tested in other animal models such as non-human primates that develop fever following SARS-CoV-2 infection [58]. 

## 5. Conclusions

We generated a cold-adapted and temperature-sensitive SARS-CoV-2 mutant TS11 that was attenuated in Syrian hamsters and induced efficient protection post virulent heterologous SARS-CoV-2 challenge. Three additional features make TS11 an attractive LAV candidate: (1) It does not contain non-conservative mutations in the receptor binding domain (RBD) of the S protein and should retain immunogenicity similar to the wildtype WA1 strain; (2) It replicates to high infectious titers in vitro which is a desirable characteristic for LAV production; and (3) It contains one mutation in the nsp16 and the deletion of partial orf7b and entire orf8 which may induce stronger innate immune responses than wildtype WA1. Having LAVs for COVID-19 is critical because LAVs could be safe, effective, and affordable for LMICs. It can be stored and transported at −20 °C but does not require −70 °C (for mRNA vaccines) due to the stability of SARS-CoV-2 [59,60]. Such a cold chain is currently used for the polio LAVs in the polio eradication campaign in many developing countries [55]. The overall attenuation and temperature sensitivity of the SARS-CoV-2 TS11 is likely a result of the combination of all the discussed mutations. More evaluation is needed to identify which mutations in the SARS-CoV-2 TS11 genome are responsible for attenuation and temperature sensitivity. A recent study showed that potent pan-sarbecovirus VN antibodies were induced in SARS-CoV survivors who were immunized with a SARS-CoV-2 mRNA vaccine [61]. Priming with a LAV followed by boosting with an S gene-based mRNA or protein vaccine may induce broader and prolonged protective immunity than the current vaccine strategies. Since the S mRNA vaccines can be updated quickly based on circulating VOCs, this vaccination strategy could be the direction for the development of pan-SARS-CoV or even pan-sarbecovirus vaccine strategies for the future. 

## Figures and Tables

**Table 1:** Mutation sites of SARS-CoV-2 TS11 compared with WA1 strain.

Nucleotide Position	WA1		TS-11		Protein
	Nucleotide	Amino Acid	Nucleotide	Amino Acid	
344	CTC	L	TTC	F	nsp1
548	ATT	I	CTT	L	
2393	GTC	V	ATC	I	
4200	ATG	M	AAG	K	nsp3
4455	GCC	A	GTC	V	
5007	ACG	T	ATG	M	
5097	ATT	N	AGT	S	
7086	ACT	T	ATT	I	
15,240	AAC	N	AAT	N	nsp12
16,411	GAT	D	AAT	N	nsp13
19,893	GAT	D	GAG	E	nsp15
20,863	CAT	H	TAT	Y	nsp16
22,120	TTC	F	TTT	F	S
22,296	CAT	H	CGT	R	
23,594–23,629		TNSPRRARSVAS	36-nt-Del	12-aa-Del	
24,000	AGC	S	ATC	I	
24,554	ACA	T	GCA	A	
26,339	GCC	A	GTC	V	E
26,571	CTT	L	TTT	F	M
26,907	CTG	L	TTG	L	
27,524	TCA	S	TTA	L	orf7a
27,807–28,177			371-nt-Del		deletion of aa 18–43 of orf7b; deletion of orf8
28,866	ACT	T	ATT	I	N

## Data Availability

The data that support the findings of this study are available in this article or from the corresponding author upon reasonable request.

## Supplementary Materials

The following supporting information can be downloaded at: https://www.mdpi.com/article/10.3390/v15010095/s1, Figure S1: Representative histopathological lesions in the lungs of Mock-C hamsters post challenge (panels A, C, and E) and TS11-C at 20 dpi as negative control (NC) hamsters (panels B, D, and F).
