# Supplementary material for: The Cold-Adapted, Temperature-Sensitive SARS-CoV-2 Strain TS11 Is Attenuated in Syrian Hamsters and a Candidate Attenuated Vaccine
Source: Viruses. 2022 Dec 29;15(1):95. doi: 10.3390/v15010095 (PMC9867033; doi:10.3390/v15010095)
Supplement: Supplementary file 1 [file viruses-15-00095-s001.zip › viruses-2107422-supplementary.pdf]

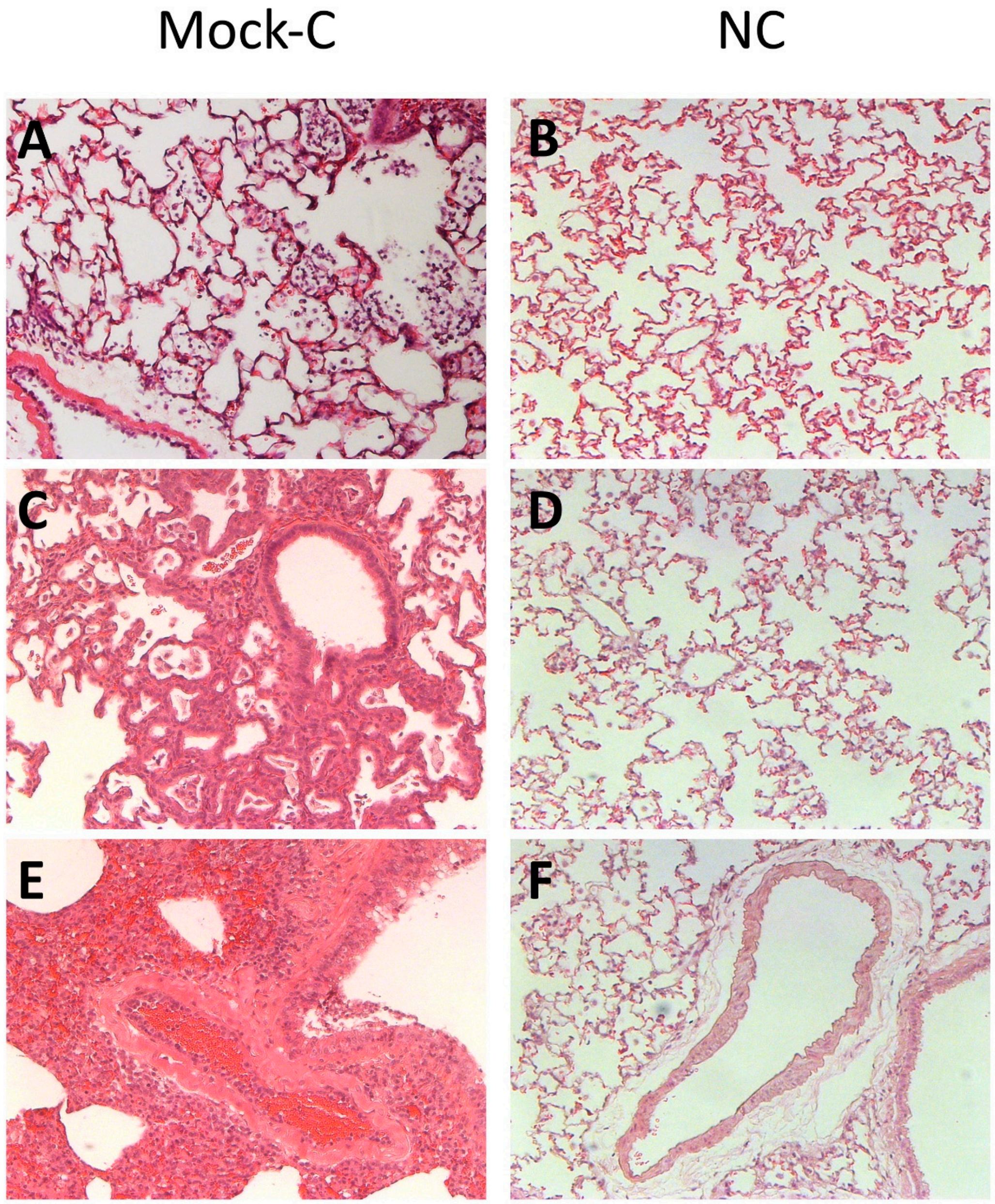

Figure S1. Representative histopathological lesions in the lungs of Mock-C hamsters post challenge (panels A, C, and E) and TS11-C at 20 dpi as negative control (NC) hamsters (panels B, D, and F). A) The hematoxylin and eosin (H&E)-stained lung section of a Mock-C hamster at 2 days post-challenge (dpc), showing moderate alveolitis. B) The H&E-stained lung section of a negative control hamster, showing normal alveoli. C) The H&E-stained lung section of a Mock-C hamster at 12 dpc, showing moderate thickening of alveolar septa. D) The H&E-stained lung section of a negative control hamster, showing normal alveoli. E) The H&E-stained lung section of a Mock-C hamster at 2 dpc, showing moderate endothelialitis or vasculitis. F) The H&E-stained lung section of a mock control hamster, showing a normal vessel. Original magnification, ×200.
